# MicroRNA-320a: an important regulator in the fibrotic process in interstitial lung disease of systemic sclerosis

**DOI:** 10.1186/s13075-020-02411-9

**Published:** 2021-01-11

**Authors:** Yiqun Li, Jing Huang, Chaojun Hu, Jiaxin Zhou, Dong Xu, Yong Hou, Chanyuan Wu, Jiuliang Zhao, Mengtao Li, Xiaofeng Zeng, Changzheng Liu, Qian Wang, Yan Zhao

**Affiliations:** 1 Department of Medical Oncology, National Cancer Center/National Clinical Research Center for Cancer/Cancer Hospital, Chinese Academy of Medical Sciences and Peking Union Medical College, Beijing, China; 2 Department of Rheumatology, Central South University Xiangya Hospital, Changsha, China; 3 Department of Rheumatology, Peking Union Medical College Hospital, Peking Union Medical College & Chinese Academy of Medical Sciences, National Clinical Research Center for Dermatologic and Immunologic Diseases (NCRC-DID), Key Laboratory of Rheumatology and Clinical Immunology, Ministry of Education, Beijing, China; 4 NHC Key Laboratory of Systems Biology of Pathogens and Christophe Mérieux Laboratory, IPB, CAMS-Fondation Mérieux, Institute of Pathogen Biology (IPB), Chinese Academy of Medical Sciences (CAMS) & Peking Union Medical College, Beijing, China

**Keywords:** Systemic sclerosis, miR-320a, Interstitial lung disease, TGF-β, IGF-1

## Abstract

**Background:**

Systemic sclerosis (SSc) is an acquired autoimmune disorder characterized by excessive accumulation of collagen and progressive tissue fibrosis. Although interstitial lung disease (ILD) complicates the majority of SSc patients and is the leading cause of death, its pathogenesis remains largely unclear. In the current study, we aimed to evaluate the role of microRNAs in SSc-ILD.

**Methods:**

miRNA expression patterns were assessed by miRNA array and real-time PCR from serum and PBMCs of SSc-ILD patients and healthy controls. Bleomycin-induced SSc-ILD mouse model was used to verify the miRNA expression in the lung tissue. The function of miRNAs in pulmonary fibroblasts was assessed using miRNA inhibitors, and mimics.

**Results:**

miR-320a was significantly downregulated in both SSc-ILD patients and mouse models. The inhibition or overexpression of miR-320a in human pulmonary fibroblasts significantly affected the protein expression of type I collagen. Luciferase reporter assay, RT-PCR, and western blot analysis identified TGFBR2 and IGF1R as direct targets of miR-320a. Upon TGF-β stimulation, the expression of miR-320a and collagen genes were significantly upregulated.

**Conclusion:**

miR-320a, together with its target genes, TGFBR2 and IGF1R, constituted a complex regulatory network, and played an important role in the fibrotic process of SSc-ILD. Investigation of more detailed mechanisms of miR-320a-mediated regulation of collagen expression may provide new therapeutic strategies for SSc-ILD.

**Supplementary Information:**

The online version contains supplementary material available at 10.1186/s13075-020-02411-9.

## Introduction

Systemic sclerosis (SSc) or scleroderma is an acquired autoimmune disorder featured by inflammation, fibrosis, and microvascular disease [1, 2]. The activation of fibroblasts and subsequent excess accumulation of extracellular matrix result in progressive fibrotic replacement of normal tissue, which ultimately leads to organ failure. Interstitial lung disease (ILD) complicates SSc in up to two third of patients and currently represents the leading cause of death in SSc [3]. However, the pathogenesis of SSc-ILD remains unclear, and the management of SSc-ILD patients remains a great challenge. MicroRNAs (miRNAs) are short (19–25 nucleotides), evolutionarily conserved, single-stranded non-coding RNAs which are involved in multiple biological processes by regulating mRNAs via cleavage or translational repression [4]. Recent evidence has indicated that miRNAs play important roles in the fibrotic process of SSc [5]. The subsequent functional studies have shown that many miRNAs act as critical regulators in fibrosis-related signaling pathways and are predicted to regulate SSc-correlated genes, for instance, collagens, integrins, and metalloproteinase [6–8].

Despite all these findings, the role of specific miRNA in the pathogenesis of SSc-ILD is not well-established. Herein, we show that miR-320a, downregulated in the serum and peripheral blood mononuclear cells (PBMCs) in SSc-ILD patients, might participate in the fibrotic process of SSc-ILD by promoting collagen synthesis through TGF-β signaling pathway. Mechanistically, miR-320a was found to be significantly downregulated in serum and PBMCs in SSc-ILD patients compared with healthy controls. Downregulated miR-320a was further verified in the lung tissue of bleomycin-induced SSc-ILD mouse models. Functional studies revealed that miR-320a regulated type I collagen expression through TGF-β signaling pathway by targeting TGFBR2 in cultured human pulmonary fibroblasts.

Our work provides insight into the molecular mechanisms through which microRNA-320a regulate collagen expression to promote the fibrotic process in SSc. Investigation of more detailed mechanisms may provide new therapeutic strategies for SSc-ILD.

## Materials and methods

### Patients and blood samples

This study was approved by the medical ethics committee of Peking Union Medical College Hospital (PUMCH) and the ethics committee of the European League Against Rheumatism Scleroderma Trial and Research Group (EUSTAR). For microRNA array analysis, PAXgene-tube blood samples were collected from 2 groups of patients. In the SSc group, there were 5 patients who satisfied the 1980 American Rheumatism Association classification criteria for SSc [9], 3 of whom were with ILDs (designated by SSc-1, SSc-2, and SSc-3) and 2 were without ILDs (designated by SSc-4 and SSc-5). In the healthy control (HC) group, there were 3 individuals who were matched to SSc patients by ages and genders (designated by HC-1, HC-2, and HC-3). Patients in the SSc group were in relative active disease status as they had not received steroid treatment for at least 2 years. For quantitative reverse transcription polymerase chain reaction (qRT-PCR) analysis, EDTA-anticoagulant blood samples were collected from 30 SSc patients who satisfied the 1980 American Rheumatism Association classification criteria for SSc (17 of them were with ILDs and 13 were without ILDs) and 16 HCs who were matched to SSc patients by ages and genders.

The PAXgene-tube blood samples were stored at − 80 °C for RNA extraction via PAXgene® blood RNA kit (Qiagen). The EDTA-anticoagulant blood samples were separated into two parts, PBMCs and plasmas. PBMCs were stored in TRIzol agents (Invitrogen) at − 80 °C for RNA extraction, and plasmas were stored in Eppendorf tubes at − 80 °C for RNA extraction. MiRNeasy serum/plasma kit (Qiagen) was used for microRNA enrichment. All the RNA/microRNA samples were kept in a − 80 °C freezer for no more than 2 days until they were transcribed into cDNAs using TaqMan MicroRNA Reverse Transcription Kit (Applied Biosystems).

### MicroRNA array and quality control

The cDNAs from PAXgene blood samples were sent to Phalanx Biotech Group for processing. These samples were purified via Nanosep 100k (Pall) and Vivaspin 500 3k (Sartorius Stedim Biotech). Purified cDNAs were examined by OD values (NanoDropTM 2000c), gel electrophoresis and capillary electrophoresis for quality control, and then labeled with Universal Linkage system (Kreatech) for hybridization on microRNA microarray (Phalanx) in NimbleGen Hybridization System. The images were scanned by the Molecular Devices AXON4000B scanner, and the figure signals were transformed to digital signals by GenePix™ 4.

### Bioinformatics and functional analysis

Differentially expressed microRNAs (DEMs) from the microarray study were analyzed through both normalized intensity comparison and cluster analysis. Normalized intensity comparison of microRNA expressions was conducted with fold change test and *t* test. In the fold change test, DEM fold changes are assumed to be within the range of 0.667 and 1.5000 (which are the 2 to the powers of range between − 0.585 and 0.585), while in the *t* test, *p* value less than 0.050 is considered to be statistically significant. Target Scan and miRanda databases are used to predict target genes of these DEMs, with the filter setting of context score < − 0.150 in the Target Scan database and *p* value < 0.005 in the miRanda database. The predicted target genes are clustered by their functions using DAVID (the Database for Annotation, Visualization and Integrated Discovery).

### Cell culture

IMR90 cells and HEK 293 cells were from the cell bank of Chinese Academy of Medical Sciences Basics Medical Science Institute (CAMSBMSI). IMR90 cells were expanded by outgrowth culture at 37 °C in a humidified atmosphere of 95% air-5% CO_2_ in Minimum Essential Media-Earle’s Balanced Salt Solution (MEM-EBSS, CAMSBMSI) supplemented with 10% heat inactivated fetal bovine serum (FBS, CAMSBMSI). HEK 293 cells were expanded by outgrowth culture at 37 °C in a humidified atmosphere of 95% air-5% CO_2_ in Dulbecco’s Modified Eagle’s Medium (DMEM, CAMSBMSI) supplemented with 10% heat inactivated fetal bovine serum (FBS, CAMSBMSI). Cells were cultured in an 80–90% confluent condition, and passages 3–5 were used for the experiments.

### RNA isolation and quantitative real-time PCR analysis

Total RNA from the fibroblasts were isolated using TRIzol RNA extraction protocol. The TaqMan® microRNA Reverse Transcription Kit (Applied Biosystems™) was used to measure the expression levels of miR-320a in a real-time PCR system analyzer (Bio-Rad IQ5), with a miR-320a specific primer. U6B small nuclear RNA (RNU6B) was used as endogenous control. cDNA was prepared by High-Capacity cDNA Reverse Transcription Kit (Applied Biosystems®) and amplified by real-time PCR with SYBR Green (SYBR Premix Ex Taq RT-PCR kit; Takara, Shiga, Japan) and appropriate primers (listed below). Expression of the GAPDH was used as endogenous control to normalize the sample data. Relative expression levels were calculated with the 2^-ΔΔCt^ method. The following primers were used:

- miR-320a—forward: 5′-CGAGCAAAAGCTGGGTTGA-3′; reverse: 5′-GTGCAGGGTCCGAGGT-3′
- U6snRNA—forward: 5′-CTCGCTTCGGCAGCACATATACT-3′; reverse: 5′-ACGCTTCACGAATTTGCGTGTC-3′

### Western blot

Samples containing equal amounts of protein were subjected to 12% SDS-PAGE and transferred to a polyvinylidene difluoride membrane (0.2 μm; Millipore, USA). After being blocked with 2% bovine serum albumin, the blots were probed with anti-TGFBR1, anti-TGFBR2, anti-IGF1 and anti-IGF1R antibody (Abcam), and anti-GAPDH antibody (ZhongShanJinQiao). After being washed with tris-buffered saline and Tween 20, the membranes were treated with horseradish peroxidase-conjugated secondary antibody (ZhongShanJinQiao) at room temperature for 1 h and visualized by enhanced chemiluminescence (Millipore, USA).

### Cell immunofluorence staining

Cells were firstly cultured on glass cover slips in 12-well plates for 1 day, then washed with PBS and fixed in 4% paraformaldehyde for 10–15 min at room temperature. Following washing, cells were then permeabilized in 0.5% Triton X-100, washed again and blocked with 3% BSA/PBS for 30 min at room temperature. The cells were then immunostained with antibodies against COL1A1 (dilution, 1:100) at 4 °C overnight. Finally, the primary antibody was detected with the Alexa Fluor® 488-conjugated donkey anti-rabbit IgG (dilution, 1:500) for 30 min at room temperature under light protection. The immunofluorescence images were viewed under a fluorescence microscope (Nikon Ti-S, Japan).

### Plasmids and constructs

The predicted binding sites within 3′UTR of human *IGF1R*, *TGFBR1*, and *TGFBR2* genes of miR-320a were amplified by PCR using human cDNA as a template. The following primers were used:

- IGF1R—forward: 5′-TCTAGATCTGGTCAACATTGTT-3′; reverse: 5′-GCGGCCGCACTGTGCTGACAAT-3′ (5535–6142, 608 nt)
- TGFBR1—forward: 5′-TCTAGAGTTATGTGGTATTCTGT-3′; reverse: 5′-GCGGCCGCTACTGTATGGGTTAC-3′ (1058–1726, 669 nt)
- TGFBR2—forward: 5′-TCTAGATGTCCATTCAAGCAGTC-3′; reverse: 5′-GCGGCCGCCTAGAGGTTCTATTT-3′ (1424–1982, 558 nt)

The PCR fragment was cloned downstream of the luciferase gene between the NheI and HindIII sites in pRL-TK (Promega). To generate 3′UTR substitution mutations in the conserved miR-320a binding site, site-directed mutagenesis of the predicted miR-320a binding sites within 3′UTR of *IGF1R*, *TGFBR1*, and *TGFBR2* genes were performed using the wild-type 3′UTR as the template. In the 3′UTR mutants, the nucleotide sequence complementary to 2–5 nt of miR-320a was mutated to the same sequence as that in miR-320a (from AGCU to TCGA).

### Luciferase reporter gene assay

Transient transfections were performed using Lipofectamine 2000 reagent (Invitrogen) according to the manufacturer’s instructions. Briefly, cells were seeded in a 24-well plate to reach the intensity of 70–80% per well for 24 h before transfection. For each well, 0.2-μg luciferase reporters containing the wild-type or a substitution mutation were cotransfected with miRNA scramble or miR-320a mimics (0.075 μg) into HEK 293 cells. As an endogeneous control, renilla luciferase reporters (0.02 μg) were also included. Forty-eight hours after transfection, the cells were collected and dual luciferase activities were measured in accordance with the manufacturer’s instructions (Promega).

### Bleomycin-induced SSc-ILD mouse model

The animal experiment was approved by the animal care and use committee in PUMCH. Six-week-old female C3H mice were continuously treated with subcutaneous injection of bleomycin at a concentration of 1 mg/ml daily for 7 weeks. Skin and lung tissues were obtained at the end of 4th week and 7th week, respectively. The removed tissues were fixed in 4% paraformaldehyde and embedded in paraffin. Sections (4 mm in thickness) were stained with H&E or Masson’s trichrome. Dermal thickness was evaluated by measuring the maximal distance between the epidermal–dermal junction and the dermal–subcutaneous fat junction at 4 different sites in each section under 100-fold magnification. Hydroxyproline (HYP) content detection in skin and lung tissue was performed in accordance with the HYP kit instructions of NanJingJianCheng Institute of Bioengineering. The absorbance value of the sample was determined by Thermo Scientific Multiskan Spectrum.

### Statistical analysis

Numerical variables with normal distribution were compared with unpaired *t* test or paired *t* test. Non-normal distribution data were compared with Wilcoxon’s rank sum test. In multiple *t* test, the false discovery rate (FDR) was controlled. Data of at least three independent experiments were expressed as mean ± SD except for the luciferase reporter gene assay, in which the relative luciferase activity between groups was presented as mean ± SEM. *p* value less than 0.05 was considered statistically significant. All analyses were performed with GraphPad Prism 7.

## Results

### miRNA expression in SSc patients with ILD

In the microarray analysis, serum of 5 SSc patients (3 with ILD and 2 without ILD) and 3 matched healthy controls (HCs) were analyzed. The detailed clinical features of these patients were presented in Additional file 1. Eighteen downregulated microRNAs and 7 upregulated microRNAs in SSc patients were identified compared with healthy controls (Table 1).

**Table 1:** Normalized intensity comparison of differentially expressed microRNAs between SSc patients and HCs

MicroRNA	Log_2_ [fold change]	*t* test *p* value	Expression
hsa-miR-3682-3p	1.068379	0.013*	Upregulation
hsa-miR-4271	1.100022	0.096	Upregulation
hsa-miR-4481	1.381003	0.060	Upregulation
hsa-miR-4498	0.87481	0.004**	Upregulation
hsa-miR-4653-3p	1.788449	0.052	Upregulation
hsa-miR-4706	1.087915	0.046*	Upregulation
hsa-miR-4764-5p	0.776084	0.045*	Upregulation
hsa-miR-1207-5p	− 0.889822	0.460	Downregulation
hsa-miR-29b-2-5p	− 0.70392	0.008**	Downregulation
hsa-miR-320a	− 0.976112	0.033*	Downregulation
hsa-miR-320b	− 0.945275	0.002**	Downregulation
hsa-miR-320c	− 0.946686	0.034*	Downregulation
hsa-miR-320d	− 0.742298	0.035*	Downregulation
hsa-miR-320e	− 0.651448	0.020*	Downregulation
hsa-miR-324-5p	− 0.911236	0.060	Downregulation
hsa-miR-362-5p	− 0.601584	0.009**	Downregulation
hsa-miR-363-5p	− 0.924108	0.105	Downregulation
hsa-miR-3656	− 0.766701	0.058	Downregulation
hsa-miR-423-3p	− 0.792722	0.038*	Downregulation
hsa-miR-423-5p	− 1.155325	0.047*	Downregulation
hsa-miR-4284	− 0.69124	0.042*	Downregulation
hsa-miR-4508	− 0.653161	0.406	Downregulation
hsa-miR-4516	− 0.600174	0.300	Downregulation
hsa-miR-500a-3p	− 0.627692	0.003**	Downregulation
hsa-miR-652-3p	− 1.043636	0.061	Downregulation

Differentially expressed microRNAs were compared by both fold change test and *t* test

*p value < 0.05

**p value < 0.01

We then compared the microRNA expressions between two sub-groups of SSc patients (patients with ILDs and those without ILDs). In the fold change test, 18 downregulated microRNAs, such as hsa-miR-29b, hsa-miR-320a, and hsa-miR-26, and 5 upregulated microRNAs were found. Target gene prediction and gene functional clustering analysis in the differentially expressed microRNAs were performed. Genes functioning in fibronectin production, cell growth, and cell death were upregulated in SSc-ILD patients compared to SSc-non-ILD patients.

### Verifying downregulated miRNAs in patients and bleomycin-induced SSc mouse model

**Fig. 1:**
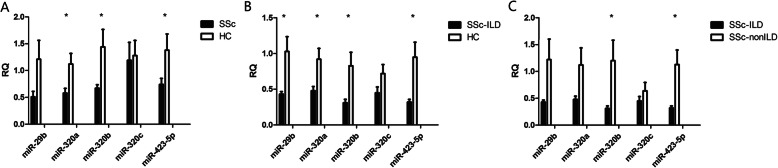
Expressions levels of microRNAs by qRT-PCR. **a** Expressions levels of microRNAs in plasma samples in SSc patients and HCs. **b** Expressions levels of microRNAs in PBMC samples in SSc patients with interstitial lung disease and HCs. **c** Expressions levels of microRNAs in PBMC samples in SSc patients with or without interstitial lung disease. *p < 0.05

According to the clustering and functional analysis, five most abundantly expressed, downregulated microRNAs (hsa-miR-29b, hsa-miR-320a, hsa-miR-320b, hsa-miR-320c, hsa-miR-423-5p) that have been reported in other diseases or related to the mechanism of pulmonary fibrosis were picked out and verified in the PBMC and plasma in 17 SSc-ILD patients, 13 SSc-non-ILD patients, and 16 matched healthy controls by real-time RT-PCR. In the plasma samples, hsa-miR-320a (*p =* 0.028), hsa-miR-320b (*p =* 0.041), and hsa-miR-423-5p (*p =* 0.029) were significantly downregulated in SSc patients compared with HCs (Fig. 1a). In the PBMC samples, hsa-miR-29b (*p =* 0.016), hsa-miR-320a (*p =* 0.021), hsa-miR-320b (*p =* 0.023), and hsa-miR-423-5p (*p =* 0.015) were significantly downregulated in SSc-ILD patients compared with HCs (Fig. 1b). In comparison of SSc-ILD patients with SSc-non-ILD patients, all five miRNAs were downregulated in SSc-ILD patients, with hsa-miR-320b (*p =* 0.043) and hsa-miR-423-5p (*p =* 0.013) reaching statistical significance (Fig. 1c). Interestingly, three (miR-320a, miR320b, and miR320c) of the five picked-out miRNAs belonged to miR320 family, and were all differentially expressed in SSc patients. Among them, the expression level of miR-320a was most strongly decreased in both the serum samples of SSc patients and the PBMC samples of SSc-ILD patients. Accordingly, we focused on miR-320a for further analysis.

**Fig. 2:**
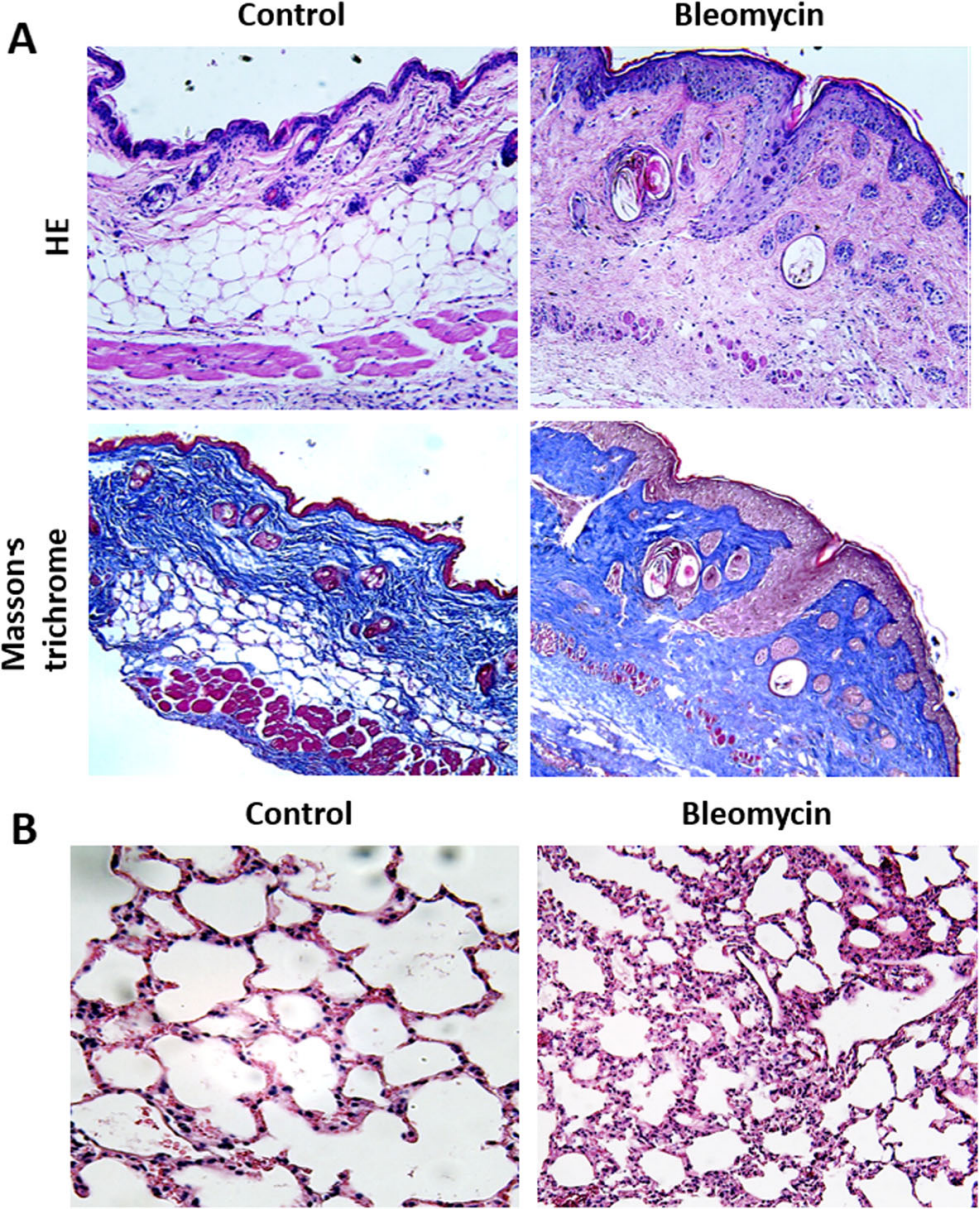
Murine model of bleomycin-induced skin and pulmonary fibrotic change. **a** Histopathology of skin lesions in bleomycin-induced mice characterized by an excessive deposition of collagens and deep fibrosis. **b** Histopathology of lung tissue in bleomycin-induced mice characterized by septal thickening, exudative inflammation, and inflammatory cell infiltration

We verified the downregulated expression of miR-320a in a bleomycin-induced SSc mouse model. The SSc mouse model was successfully induced by continuously subcutaneous injection of bleomycin at a concentration of 1 mg/ml to C3H mice for 7 weeks. Inflammatory cell infiltration could be seen in both skin and lung tissues of the mice (Fig. 2). The expression level of miR-320a was significantly downregulated in the lung tissues of SSc model mice at the end of 7 weeks treatment (RQ, 722.02 ± 150.29 vs. 1979.21 ± 1161.50; *p =* 0.029). mRNA microarray identified 1144 genes in total that significantly upregulated in the model mice group, most of which were involved in inflammation, immune response, and fibrosis.

### miR-320a regulates collagen expression

One of the most prominent features of SSc-ILD is the accumulation of collagen in the extracellular matrix, which leads to fibrosis eventually. Therefore, we hypothesized that the altered miR-320a level would have an effect on collagen expression. According to our hypothesis, the overexpression of miR-320a in pulmonary fibroblasts would decrease collagen levels, whereas downregulation of miR-320a would induce an SSc-like phenotype, with collagen overexpression. The lung tissue of SSc-ILD patients is hard to retrieve due to ethical concern, and normal human pulmonary fibroblast cell line, the IMR90 cells, were used instead in our study.

**Fig. 3:**
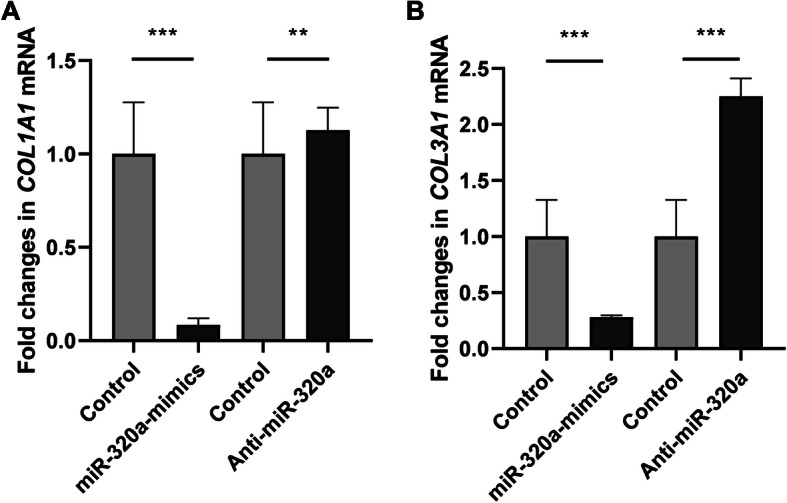
Expression of collagen genes were altered following overexpression and knockdown of miR-320a. Normal human pulmonary fibroblasts were transfected with miR-320a mimics, miR-320a inhibitors, and scramble as control. **a** mRNA levels for *COL1A1* decreased after miR-320a overexpression as compared with the control group while the levels increased after knockdown of miR-320a. **b** mRNA levels for *COL3A1* decreased after miR-320a overexpression as compared with the control group while the levels increased after knockdown of miR-320a. Experiments were repeated at least 3 times. Values are means ± SD. **p < 0.01 versus scramble controls. ***p < 0.001 versus scramble controls

**Fig. 4:**
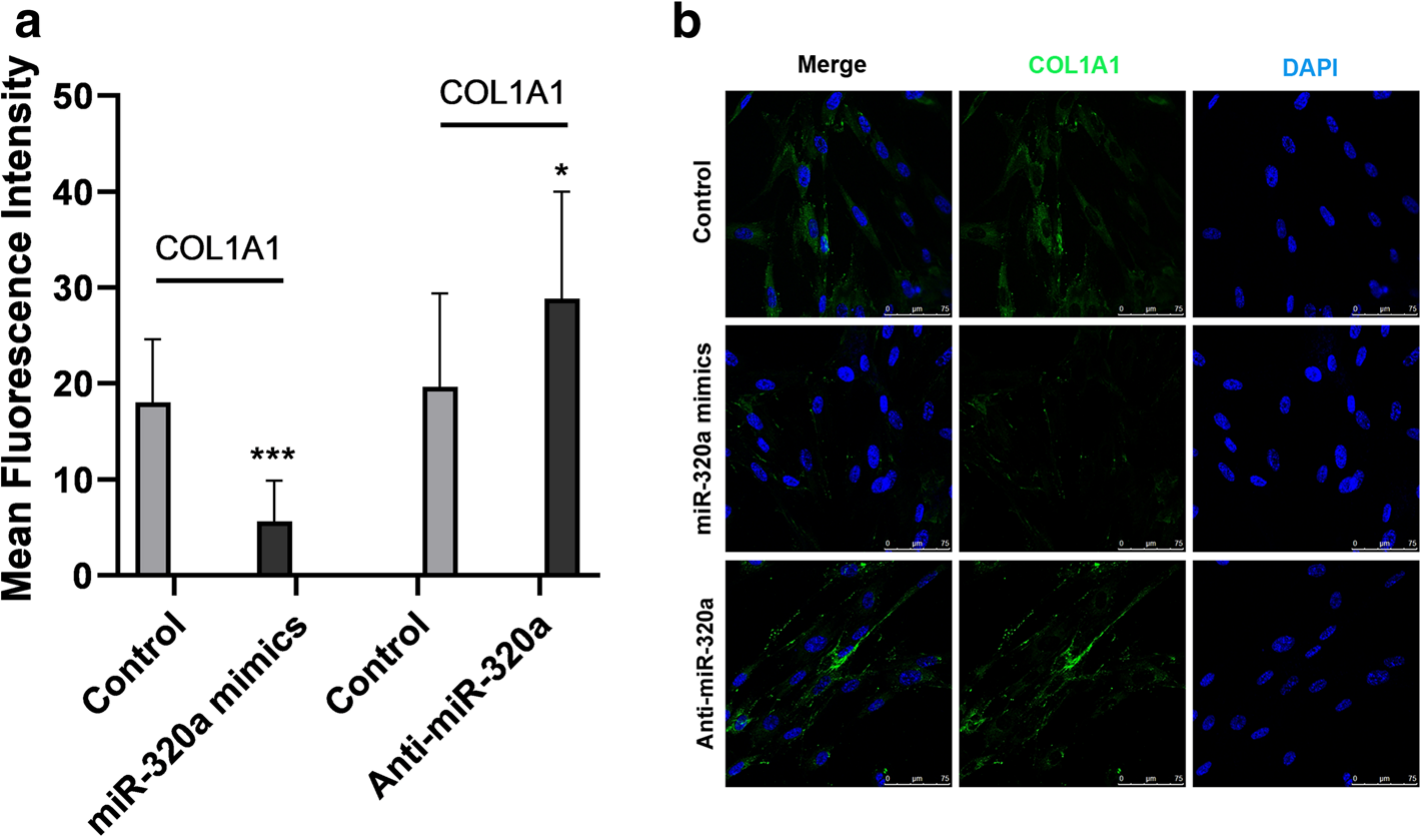
Expression of collagen genes were altered following overexpression and knockdown of miR-320a. Normal human pulmonary fibroblasts were transfected with miR-320a mimics, miR-320a inhibitors, and scramble as control. Transfected cells were fixed, permeabilized, blocked, immunostained, and subjected to immunofluorescence. Nuclei were stained with DAPI (blue channel), and COL1A1 was stained with an anti-COL1A1 antibody (green channel). **a** Images were acquired by confocal microscopy at an original magnification of × 10. Scale bar, 10 μm. **b**, **c** Quantitized immunofluorescence intensity of type I collagen in the miR-320a overexpression group was dramatically decreased compared to the control group, whereas the intensity in the miR-320a downregulation group was significantly increased. Experiments were repeated at least 3 times. Values are means ± SD. *p < 0.05 versus scramble controls. ***p < 0.001 versus scramble controls

In support of our notion, overexpression of miR-320a in IMR90 cells by transfecting with miR-320a mimics decreased the mRNA levels of *COL1A1* and *COL3A1* (the ɑ chain of type I and III collagen, thus representing the expression of type I and III collagen) significantly (*p <* 0.001) (Fig. 3a), while knockdown of miR-320a using anti-miR-320a significantly increased the mRNA levels of type I and III collagen (*p <* 0.01, *p <* 0.001) (Fig. 3b). Further cell immunofluorescence revealed that the quantitized immunofluorescence intensity of collagen I in the miR-320a overexpression group was dramatically decreased compared to the control group (*p <* 0.001) (Fig. 4a), whereas the intensity in the miR-320a downregulation group was significantly increased (*p <* 0.05) (Fig. 4b).

Altogether, our data indicates that the expression of collagen genes were negatively regulated by miR-320a in IMR90 cells.

### *TGFBR2* and *IGF1R* are direct functional targets of miR-320a

**Fig. 5:**
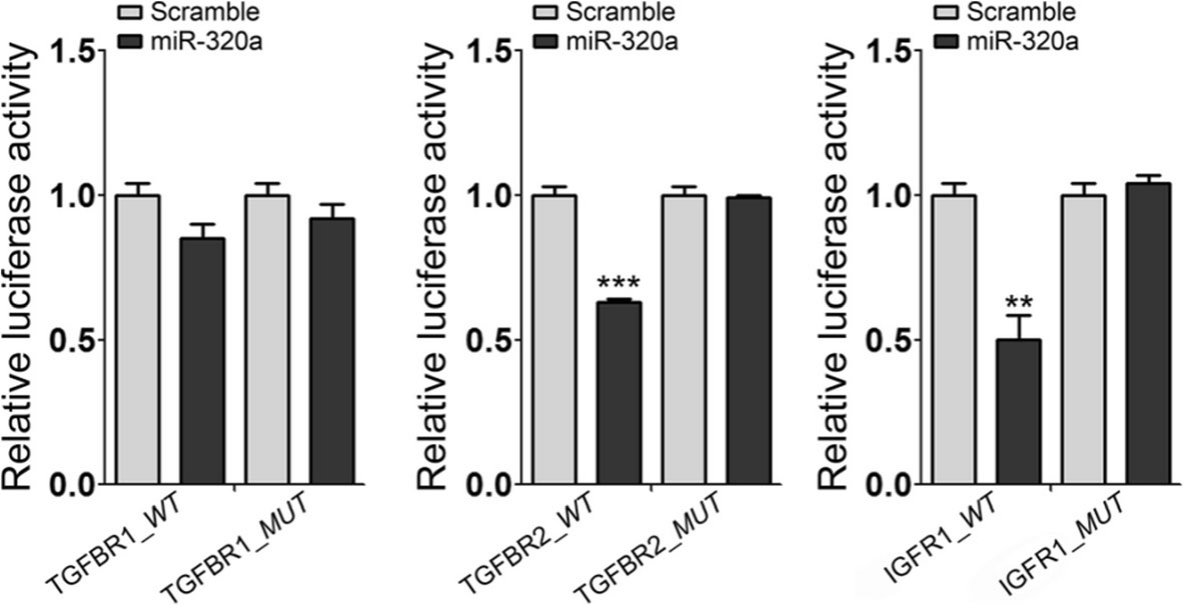
*TGFBR2* and *IGF1R* were directly regulated by miR-320a. The construction of luciferase reporter gene system: wild-type (WT) contains part of the 3′UTR of *TGFBR1*, *TGFBR2*, and *IGF1R* with binding sites for miR-21, and the mutant type (MUT) contains a deletion mutation in the predicted miR-320a seeding sequence in the 3′UTR of *TGFBR1*, *TGFBR2*, and *IGF1R*. Increasing the amounts of the miR-320a by transfection of miR-320a mimics represses the luciferase activity of *TGFBR2* and *IGF1R* 3′UTR-WT, but has no influence on the *TGFBR1* 3′UTR-WT and the mutants. Experiments were repeated at least 3 times. Values are means ± SEM. **p < 0.01 versus scramble controls. ***p < 0.001 versus scramble controls

We next aimed to clarify the possible regulatory mechanism(s) by which miR-320a affects collagen expression. Bioinformatics analysis (described in the “Materials and methods” section) was used in our study, and overlapped predicted profibrotic targets of miR-320a were included. Genes known to influence pathological fibrosis were preferentially selected to yield *TGFBR1*, *TGFBR2*, and *IGF1R*. To assess whether *TGFBR1*, *TGFBR2*, and *IGF1R* are the direct targets for miR-320a, we generated a luciferase reporter gene system by cloning a part of the 3′UTR of *TGFBR1*, *TGFBR2*, and *IGF1R* with respective binding sites for miR-320a into the pRL-TK reporter vector. Analogous 3′UTR mutant clones that contain a substitution mutation in the predicted miR-320a binding sequences were prepared. In the reporter gene assay, cotransfection of HEK 293 cells with miR-320a mimic and the luciferase reporter plasmid containing the 3′UTR of *TGFBR2* and *IGF1R* decreased the relative luciferase activity compared with cells transfected with scrambled controls and plasmid, whereas cotransfection with miR-320a mimic and the luciferase reporter plasmid containing the *TGFBR2* and *IGF1R* 3′UTR mutant clones did not show significant effects. Significantly decreased luciferase activity was not identified in HEK 293 cells cotransfected with miR-320a mimic and the luciferase reporter plasmid containing the 3′UTR of *TGFBR1* (Fig. 5)*.* Therefore, the expressing vectors containing wild-type, but not mutant *TGFBR2* and *IGF1R* 3′UTR, were subject to regulation by miR-320a, which indicated that *TGFBR2* and *IGF1R* were directly regulated by miR-320a.

**Fig. 6:**
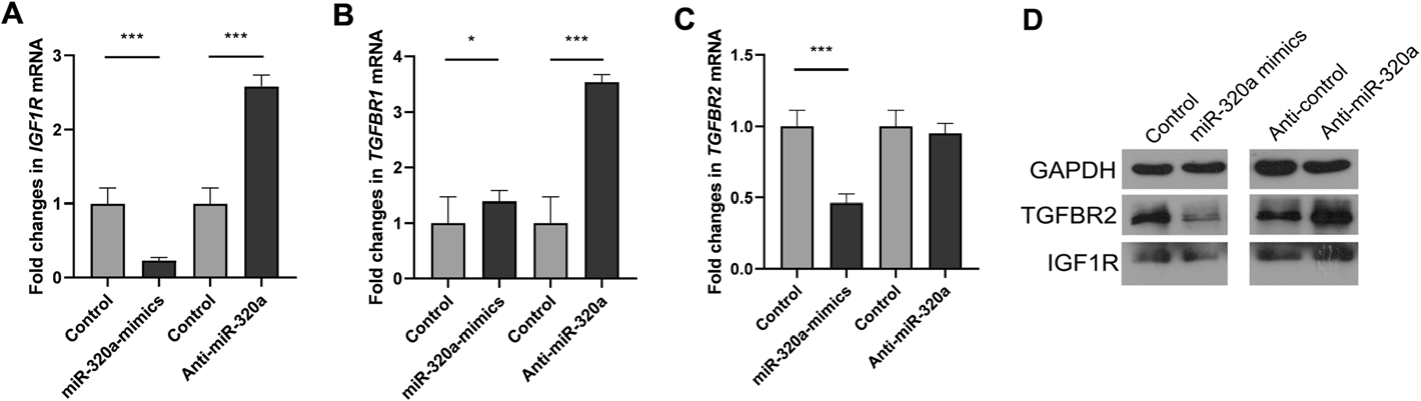
Expression of *TGFBR2* and *IGF1R* were altered following overexpression and knockdown of miR-320a. Normal human pulmonary fibroblasts were transfected with miR-320a mimics, miR-320a inhibitors, and scramble as control. **a**–**c** mRNA levels of *TGFBR1*, *TGFBR2*, and *IGF1R* were determined by SYBR Green real-time polymerase chain reaction analysis. **d** Protein was analyzed by western blot. Experiments were repeated at least 3 times. Values are means ± SD. *p < 0.05 versus scramble controls. **p < 0.01 versus scramble controls. ***p < 0.001 versus scramble controls

We next explored the functional effects of altered miR-320a expression on *TGFBR2* and *IGF1R* in cultured pulmonary fibroblasts. As expected, transfection with miR-320a mimics in IMR90 cells decreased the expression of *TGFBR2* and *IGF1R* both on mRNA and protein levels, as analyzed by RT-PCR and western blotting (Fig. 6). Thus, altered miR-320a regulated the expression of *TGFBR2* and *IGF1R* in IMR 90 cells.

### Investigate the relationship between miR-320a, TGF-β signaling pathway, and collagen expression

**Fig. 7:**
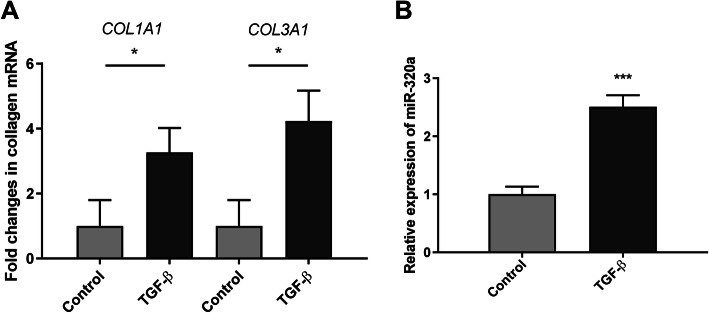
The expression levels of miR-320a and collagen genes after TGF-β stimulation. **a** mRNA levels of *COL1A1* and *COL3A1* were determined by SYBR Green real-time polymerase chain reaction analysis. **b** Relative levels of miR-320a were analyzed by real-time PCR. Experiments were repeated at least 3 times. Values are means ± SD. *p < 0.05 versus scramble controls. ***p < 0.001 versus scramble controls

We then tried to clarify the mechanisms that underlie the regulation between miR-320a, TGF-β signaling pathway, and collagen expression. We investigated the effect of exogenous TGF-β stimulation in IMR90 cells. The expression of miR-320a, COL1A1, and COL3A1 mRNA was significantly upregulated after exogenous TGF-β stimulation (Fig. 7).

## Discussion

The present study showed that miR-320a levels were associated with SSc-ILD. In model systems, we further revealed that miR-320a was involved in the regulation of biological pathways relevant to SSc-ILD. miR-320a, together with its target genes TGFBR2 and IGF1R, constituted a complex network in the fibrotic process.

In this study, we detected a constitutive downregulation of miR-320a in serum and peripheral blood mononuclear cells in SSc-ILD patients and in the lung tissue of bleomycin-induced SSc-ILD mouse models. Our study is the first, to our knowledge, to demonstrate the miR-320a downregulation in SSc-ILD patients. Previous data on miR-320a mainly focused on cancer and diabetes. miR-320a was reported to inhibit cell proliferation, migration, and invasion in different types of cancer [10, 11] and regulate angiogenesis on endothelial cell and induce target organ damages in diabetes [12, 13].

To further explore the association between miR-320a and SSc-ILD, we hypothesized that altered miR-320a level might have an effect on collagen expression, and the hypothesis was confirmed by the fact that the expression of collagen genes were negatively regulated by miR-320a at both mRNA and protein levels. Thus, we found the miRNA–mRNA interactions: miR-320a and collagen, suggesting the role of miR-320a in the regulatory mechanisms of extracellular matrix metabolism. This was in accordance with previous findings. Zhang et al. found that miR-320a could suppress chondrocyte collagen degradation via targeted inhibition of β-catenin [14]. Meng et al. reported that miR-320a regulated MMP-13 expression in chondrogenesis [15]. However, the role of miR-320a in regulating collagen expression in pulmonary fibroblasts was not investigated.

To explain the underlying mechanisms through which miR-320a regulates collagen, we performed biocomputational target gene prediction. Luciferase reporter gene assay determined *TGFBR2* as a direct target of miR-320a. Overexpression of miR-320a in pulmonary fibroblasts significantly inhibited *TGFBR2* mRNA and protein expression while inhibition of miR-320a significantly increased the expression of *TGFBR2*. TGFBR2 is a transmembrane serine/threonine kinase. Upon stimulation, TGFBR2 binds to TGF-β factor first, then activates TGFBR1, forming a heterodimeric complex to bind to TGF-β. This receptor/ligand complex phosphorylates downstream proteins, which ultimately results in regulation of a series of genes involved in fibrosis, such as collagens and fibronectin [16].

The TGF-β pathway plays critical roles in fibrosis, particularly in SSc-ILD. The downstream activation of the canonical SMAD pathway (Smad2/3, 4) causes the expression of extracellular matrix proteins. Via non-canonical pathways (e.g., SMAD1, ERK1/2), TGF-β is associated with profibrotic response and induction of myofibroblast differentiation [17]. Stimulation of autocrine TGF-β signaling was also reported in SSc fibroblasts. The pro- or anti-fibrotic role has been recognized for selected microRNAs that target factors downstream or upstream of TGF-β, such as miR-29, one of the most important miRNAs involved in TGF-β signaling pathway and collagen production, miR-21, miR-221/222, and miR-155 [6–8, 18].

The findings that miR-320a regulated both collagen expression and TGFBR2 suggest that miR-320a might modulate collagen expression through TGF-β signaling pathway via targeting TGFBR2. Therefore, we went on to investigate the effect of exogenous TGF-β stimulation in pulmonary fibroblasts. The expression of miR-320a and the collagen genes were all significantly upregulated after exogenous TGF-β stimulation, suggesting that in normal pulmonary fibroblasts, miR-320a might inhibit fibrogenic activation via overexpression upon TGF-β stimulation. In SSc patients, however, the level of miR-320a decreased due to some unknown pathological changes. The decreased miR-320a levels helped to promote TGF-β-related fibrotic process and eventually led to tissue fibrosis. Since miRNAs function as epigenetic modifications, we speculate that the decreased miR-320a levels might be a result of epigenetic alterations like epigenetic “silencing.” Further investigations might include determining the methylation or acetylation status of miR-320a in SSc-ILD patients.

Last but not least, we demonstrated that miR-320a directly regulated IGF1R in pulmonary fibroblasts. Guo et al. also reported the same finding in glioma cells [19]. IGF1R is the respective receptor of IGF1 signaling pathway, functioning in cell proliferation, differentiation, glucose homeostasis, metabolism, etc. [20]. IGF-1 has also been implicated in pathological fibrosis states. Elevated serum IGF-1 level was reported in those with more severe skin involvement and pulmonary fibrosis in SSc patients. In bleomycin-induced lung injury mouse models, IGF-1 mRNA was significantly increased in pulmonary fibrosis, and IGF-1 overexpression exacerbated the fibrotic process [21, 22]. Sequential expression of IGF-1 and TGF-β was found to synergistically aggravate fibrosis, and upregulation of IGF-1 via TGF-β in myofibroblasts was documented in the fibrotic lung tissue in advanced idiopathic pulmonary fibrosis (IPF) [23]. Collectively, these findings imply a causal pathway of miR-320a in SSc-ILD by revealing that in normal pulmonary fibroblasts, miR-320a participated in the fibrotic process through coordinating a wide spectrum of profibrotic molecules like TGF-β and IGF1 pathway mediators. miR-320a may represent a potential agent for biological strategy, and maintaining normal miR-320a levels may be beneficial in SSc-ILD patients.

In conclusion, this is the first evidence supporting the association of miR-320a levels with SSc-ILD patients and the identification of a fibrotic role of miR-320a in model systems. miR-320a, together with its target genes, TGFBR2 and IGF1R, constituted a complex regulatory network, and played an important role in the fibrotic process of SSc-ILD. Investigation of more detailed mechanisms of miR-320a-mediated regulation of collagen expression may provide new therapeutic strategies for SSc-ILD.

## Supplementary Information

**Additional file 1.** Detailed clinical features of SSc patients included in the microarray analysis.

## Data Availability

The datasets used during the current study are available from the corresponding author on reasonable request.
