# Supplementary material for: MicroRNA-320a: an important regulator in the fibrotic process in interstitial lung disease of systemic sclerosis
Source: Arthritis Res Ther. 2021 Jan 11;23:21. doi: 10.1186/s13075-020-02411-9 (PMC7802184; doi:10.1186/s13075-020-02411-9)
Supplement: Supplementary file 1 — Additional file 1. Detailed clinical features of SSc patients included in the microarray analysis. [file 13075_2020_2411_MOESM1_ESM.docx]

**Additional file 1**

Additional file 1. Detailed clinical features of SSc patients included in the microarray analysis.

| **Group** | **No.** | **Age (years)** | **Sex** | **SSc subset** | **Disease duration**  **(years)** | **mRSS** | **Anti-Scl-70** | **Anti-**  **centromere antibody** |
| --- | --- | --- | --- | --- | --- | --- | --- | --- |
| SSc-ILD | 1 | 48 | Female | lcSSc | 7 | 6 | positive | negative |
| SSc-ILD | 2 | 41 | Female | lcSSc | 3 | 12 | positive | negative |
| SSc-ILD | 3 | 36 | Female | dcSSc | 1 | 16 | negative | negative |
| SSc-nonILD | 1 | 18 | Female | dcSSc | 3 | 12 | positive | negative |
| SSc-nonILD | 2 | 48 | Female | dcSSc | 3 | 13 | negative | negative |

SSc: systemic sclerosis; mRSS: modified Rodnan skin score; ILD: interstitial lung disease
